# A chromosome-level genome assembly for the smoky rubyspot damselfly (*Hetaerina titia*)

**DOI:** 10.1093/jhered/esad070

**Published:** 2023-11-21

**Authors:** Christophe W Patterson, Erandi Bonillas-Monge, Adrian Brennan, Gregory F Grether, Luis Mendoza-Cuenca, Rachel Tucker, Yesenia M Vega-Sánchez, Jonathan Drury

**Affiliations:** Department of Biosciences, Durham University, Durham, United Kingdom; Department of Biosciences, Durham University, Durham, United Kingdom; Department of Biosciences, Durham University, Durham, United Kingdom; Department of Ecology & Evolutionary Biology, University of California, Los Angeles, California, LA, United States; Facultad de Biología, Universidad Michoacana de San Nicolás de Hidalgo, Morelia, México; NERC Environmental Omics Facility, University of Sheffield, Sheffield, United Kingdom; Instituto de Investigaciones en Ecosistemas y Sustentabilidad, Universidad Nacional Autónoma de México, Morelia, México; Department of Biosciences, Durham University, Durham, United Kingdom

**Keywords:** aquatic insect, Calopterygidae, comparative genomics, long-read sequencing, Odonata, Omni-C, PacBio, riparian, Zygoptera

## Abstract

Smoky rubyspot damselflies (*Hetaerina titia* Drury, 1773) are one of the most commonly encountered odonates along streams and rivers on both slopes of Central America and the Atlantic drainages in the United States and southern Canada. Owing to their highly variable wing pigmentation, they have become a model system for studying sexual selection and interspecific behavioral interference. Here, we sequence and assemble the genome of a female smoky rubyspot. Of the primary assembly (i.e. the principle pseudohaplotype), 98.8% is made up of 12 chromosomal pseudomolecules (2N = 22A + X). There are 75 scaffolds in total, an N50 of 120 Mb, a contig-N50 of 0.64 Mb, and a high arthropod BUSCO score [C: 97.6% (S: 97.3%, D: 0.3%), F: 0.8%, M: 1.6%]. We then compare our assembly to that of the blue-tailed damselfly genome (*Ischnura elegans*), the most complete damselfly assembly to date, and a recently published assembly for an American rubyspot damselfly (*Hetaerina americana*). Collectively, these resources make *Hetaerina* a genome-enabled genus for further studies of the ecological and evolutionary forces shaping biological diversity.

## Introduction

Given their easily observable behavior during adult life stages, odonates (Order Odonata, containing both dragonflies and damselflies) have emerged as model systems for many questions in ecology and evolutionary biology (Corbet 1999; Córdoba-Aguilar et al. 2023). Yet, genomic resources for aquatic insects are underrepresented relative to resources for other terrestrial insects (Hotaling et al. 2020). There are currently only 10 odonate species with de novo assemblies present on GenBank, of which six are classified as chromosome-level assemblies and four are scaffolds.

Rubyspot damselflies (genus *Hetaerina*), in particular, have been widely studied among investigators interested in sexual selection (e.g. Grether 1996; Córdoba-Aguilar et al. 2009), as well as in understanding the causes and consequences of behavioral interference between species (e.g. Anderson and Grether 2010; Drury et al. 2019; Grether et al. 2020). Recently, a genome assembly of *Hetaerina americana* was published (Grether et al. 2023), enabling genomic research on this group of charismatic insects. However, because the best estimate for the crown age for the *Hetaerina* is 36.2 million years ago (Mya) and phylogenetic analyses demonstrate that the clade is paraphyletic and requires taxonomic revision (Standring et al. 2022), more genomic resources beyond *H. americana* are needed to fully resolve species relationships and support genomic research on this taxon. Moreover, although the *H. americana* assembly provides a high-quality set of genomic resources, the scaffolds are not fully resolved into chromosomes; estimates from *Calopteryx* spp. suggest that members of Calopterygidae have 12 pairs of autosomes and an XO sex-determination system: females carry two X chromosomes, and male carry a single X chromosome (Kuznetsova and Golub 2020; Kuznetsova et al. 2021), but the *H. americana* assembly comprises over 1,500 scaffolds.

**Fig. 1.**
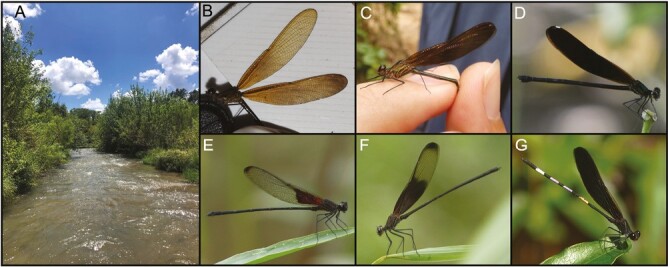
A) An example photograph of a typical habitat where smoky rubyspot damselflies (*Hetaerina titia*) are found (Medina River in Castroville, Texas, United States). B and C) The female *H. titia* individual collected in Santa María Huatulco, Oaxaca, Mexico was used to construct the draft genome. D–G) Examples of variation of wing pigmentation in D) a highly melanized female (cf. panel C), and E–G) males. All photos by Jonathan Drury.

One of the most widely studied *Hetaerina* species, smoky rubyspot damselflies (*Hetaerina titia*), currently lacks genomic resources. Smoky rubyspots damselflies are the most phenotypically distinct of rubyspot damselflies—both males and females possess melanin pigment in their wings (Fig. 1) that distinguish them from the relatively transparent wings typical of other rubyspot damselflies. However, the extent of dark pigmentation varies both seasonally and geographically (Drury et al. 2015; Drury, Barnes, et al. 2019). Based on this extensive variation, *H. titia* was once divided into two species, *H. titia* and *H. tricolor* (Johnson 1963), though current taxonomic consensus treats *H. titia* as one highly variable lineage (Garrison 1990; Drury et al. 2015).

Like other rubyspot damselflies, *H. titia* occurs along streams and rivers, with males defending mating territories along the banks and females descending from higher hunting perches to the river generally only to mate and oviposit. However, territorial males tend to perch higher and in slower parts of streams than sympatric congeners (McEachin et al. 2022). Moreover, *H. titia* exhibits the largest latitudinal range of any *Hetaerina* species, stretching from Panama in the south to Canada in the north.

Here, we report the first genome assembly for *H. titia*, based on long-read sequence data from a female collected in Oaxaca, Mexico and Omni-C sequencing from a female collected in Chiapas, Mexico. We also present analyses comparing our assembly to the recently published genome of *H. americana* (Grether et al. 2023) and to the highest quality zygopteran (damselfly) genome assembly of *Ischnura elegans* (Price et al. 2022).

## Methods

### Biological materials

A wild female *H. titia* (Fig. 1b and c) was collected from Santa María Huatulco, Oaxaca, Mexico (15.74, −96.298) in June 2021 and submerged in RNAlater (Invitrogen) for HiFi sequencing. Another female *H. titia* was collected from Nuevo Milenio Santa Cruz la Central, Chiapas, Mexico (15.76, −93.30) in April 2023 and submerged in RNAlater for Omni-C scaffolding. We note that, given the XO sex-determination system of Calopterygidae, all regions of the genome are sequenced by choosing female specimens. Specimen collection was conducted under permit SGPA/DGVS/04421/21, issued by the Mexican Secretaría de Medio Ambiente y Recursos Naturales (SEMARNAT).

### Nucleic acid library preparation

High molecular weight (HMW) DNA extraction was carried out at the Natural Environmental Research Council (NERC) environmental ‘omics facility in Sheffield, United Kingdom. Upon receipt, the tissue samples were immediately removed from RNAlater and stored in SET buffer (75 mM NaCl, 25 mM EDTA pH 8, 20 mM Tris–HCl pH 7.5) at −80 °C. Fresh lysis buffer and Proteinase K solution were prepared as described in “10 x Genomics DNA Extraction from Single Insects” (https://support.10xgenomics.com/de-novo-assembly/sample-prep/doc/demonstrated-protocol-dna-extraction-from-single-insects 10 × Genomics) with modifications. Briefly, the whole damselfly was added to a lysis buffer of 10 mM Tris–HCl, 400 mM NaCl, 100 mM EDTA (pH 8.0), 10% SDS, and supplemented with 10 nM dithiothreitol (DTT). A Proteinase K solution containing 2 mg/ml of Proteinase K, 1% SDS, and 4 mM EDTA (pH 8.0) was added, and the sample was then homogenized in the lysis buffer by a pestle. The sample was left to digest overnight at 37 °C.

The DNA was precipitated by adding 5 M NaCl and 100% ethanol, and, following centrifugation, the resulting pellet was washed twice with 70% ethanol. The DNA was resuspended in elution buffer [Pacific Biosciences (PacBio), Cat. #101-633-500] and left to elute at room temperature for 48 h. The DNA was purified and concentrated twice with 0.45 × AMPure beads (PacBio, Cat. #100-265-900), to ensure samples were not contaminated with RNA, which can inhibit sequencing. To remove degraded DNA fragments (<9 kb), and contamination such as pigment present in the tissue, the DNA sample was loaded onto the Blue Pippin (Sage Science), using a 0.75% Agarose Cassette (Sage Science, Cat. #BLF7510) and 0.75% DF Marker S1 high-pass 6 to 10 kb v3 cassette definition file. Fragments larger than 9 kb were collected, and smaller fragments were discarded. The size selected DNA sample was purified using 0.45× AMPure beads.

Purified DNA was quantified using a Qubit 3 Fluorometer (Invitrogen Q33216), its purity assessed using 260/280 and 260/230 ratios determined using a NanoDrop 8000 Spectrophotometer (Thermo Scientific ND-8000-GL), and finally analyzed on a Femtopulse system to accurately size the fragments present within the sample (Agilent Technologies, Santa Clara, California).

### DNA sequencing

HiFi Library Preparation and Sequencing was completed at the NERC environmental ‘omics facility in Liverpool, United Kingdom. A HiFi SMRTbell library was constructed using the SMRTbell Express Template Prep Kit v2.0 with Low DNA Input (PacBio Cat. #100-938-900) according to the manufacturer’s instructions. The HiFi SMRTbell library was sequenced using one SMRT Cell 8M, Sequel II sequencing chemistry 2.0, and 10-h movies on a PacBio Sequel II sequencer.

### Nuclear genome assembly

Circular consensus sequencing (CCS) reads with a quality score lower than 20QV were removed. PacBio HiFi adapters were screened and removed using HiFiAdapterFilt v2.0 (Sim et al. 2022). To estimate genome size and sequencing error rate, we used *jellyfish v2.3.0* (Marçais and Kingsford 2011) to count k-mers using *k* = 21 and then ported the data into *genomescope v2* (Ranallo-Benavidez et al. 2020). The *genomescope* genome size estimate was used to establish the most appropriate parameters for genome assembly. Genome assembly was then conducted using the program *Hifiasm v.0.16* (Cheng et al. 2021, 2022) using default parameters. *Hifiasm* outputs two assemblies: a primary and alternative assembly consisting of each haplotype of the diploid genome. We ran *purge_dups v1.2.6* (Guan et al. 2020) on the primary assembly to identify duplicated regions of the assembly from either unidentified haplotypes or contig overlaps. We then removed these duplicates from the primary assembly, transferred them to the alternate assembly, and then ran *purge_dups* on the alternate assembly. We then screened the assemblies for contamination using *blastn* and *blobtoolkit2*/3.1.6 (Table 1) (Challis et al. 2020).

**Table 1.** Assembly pipeline with software and version, as well as the options chosen where they differ from default of each software.

Assembly stage	Software and version	Options
Assembly		
Filtering PacBio HiFi adapters	HiFiAdapterFilt v2.0	*-l 34 -t 64 -m 80*
K-mer counting	jellyfish v2.3.0	*k* = 21
Estimation of genome size and heterozygosity	GenomeScope v1.0^a^	
De novo assembly (contiging)	HiFiasm v0.16.1	
Remove low coverage, duplicated contigs	purge_dups v1.2.6	purge_dups -2 -T cutoffs, get_seq -e
Organelle assembly		
Mitochondrial genome	mitohifi v2.2	-c -o 5 -f MK722304.1 -g MK722304.1
	mitos2	
Genome quality assessment		
Genome completeness	BUSCO v5.3.2	-m genome -l insect_obd10/arthropoda_obd10
Contamination	Blastn (blobtoolkit2/3.1.6)	-outfmt “6 qseqid staxids bitscore std” -max_target_seqs 10 -max_hsps 1 -evalue 1e-25
Assembly metrics	quast/5.2.0	

^a^Output archived at http://qb.cshl.edu/genomescope/analysis.php?code=JRQJM0S1JoMbmflwyqd1.

### Dovetail Omni-C library preparation and sequencing

To further increase the contiguity of the assembly, Omni-C sequencing was conducted by Dovetail Genomics (Scotts Valley, United States). Omni-C sequencing utilizes the proximate physical distance between adjacent stretches of DNA in the nucleus to generate a map of physically linked DNA. Following standard manufacturer’s protocol, formaldehyde was used to fix the chromatin within the nucleus and then the chromatin was extracted. DNAse I was used to digest the fixed chromatin and then chromatin ends were repaired and ligated to a biotinylated bridge adapter followed by proximity ligation of adapter-containing ends. After proximity ligation, crosslinks were reversed, and the DNA purified. Purified DNA was treated to remove biotin that was not internal to ligated fragments. Sequencing libraries were generated using NEBNext Ultra enzymes and Illumina-compatible adapters. Biotin-containing fragments were isolated using streptavidin beads before PCR enrichment of each library. The library was sequenced on an Illumina HiSeqX platform to produce approximately 30× sequence genome coverage. Scaffolds were constructed using HiRise with MQ >50 reads (Putnam et al. 2016).

To construct a scaffolded genome assembly, we used the uncontaminated primary contig assembly and Dovetail Omni-C library reads as input data for HiRise (Putnam et al. 2016). Dovetail Omni-C library sequences were aligned to the draft input assembly using *bwa* (Li and Durbin 2009). The separations of Dovetail Omni-C read pairs mapped within draft scaffolds were analyzed by HiRise to produce a likelihood model for genomic distance between read pairs, and the model was used to identify and break putative misjoins, to score prospective joins, and make joins above a threshold.

### Quality assessment and assembly metrics

To estimate genome completeness and accuracy, we calculated the BUSCO v5.3.2 (Manni et al. 2021) score of our assemblies using the lineage datasets *insecta_odb10* and *arthropoda_odb10.* Basic genome assembly metrics were calculated using *quast/5.2.0* (Mikheenko et al. 2018). To compare our assembly’s completeness and contiguity in relation to other available odonate assemblies, we calculated BUSCO scores (using insecta_ob10 and arthropoda_ob10) and NGx metrics for 10 odonate genomes on GenBank. We excluded assemblies where there was an obvious higher-quality genome present for the same species.

### Mitochondrial genome assembly

We assembled the mitochondrial genome of *H. titia* using the *mitohifi v2.2* (Uliano-Silva et al. 2023) pipeline, which identified the mitochondrial genome of the confamilial species *Matrona basilaris* (GenBank: MK722304.1) as a reference—the closest annotated mitogenome to *H. titia* available on NCBI. The mitochondrial annotation was conducted using *mitos2* (Donath et al. 2019). Any identified contigs from the draft nuclear genome that were smaller than the draft mitochondrial genome and had a >99% match to the mitochondrial genome, using BLAST+ (Camacho et al. 2009), were then removed from the primary (*n* = 1) and alternate (*n* = 0) assemblies using a custom R script and *blobtools* (Challis et al. 2020).

### Comparison with I. elegans and H. americana

We visualized the synteny between the *H. titia* primary assembly with that of the chromosome-level genome of *I. elegans* using a Circos Assembly Consistency plot built using the pipeline JupiterPlot (Chu 2018), and a custom R script for final visualization using the *circulize v0.4.15* package (Gu et al. 2014) (Table 2). We also visualized the correspondence between *H. titia* vs. the draft genome of *H. americana* (Grether et al. 2023) and the genome of *I. elegans* (Price et al. 2022) vs. *H. americana.* We used LASTZ (Harris 2007) to align each of the genomes and calculated the overall percentage of genomic similarity between *H. titia* and *I. elegans*, *H. americana* and *I. elegans*, and *H. titia* and *H. americana* using the total aligned length divided by the total number of mismatches.

**Table 2.** Genome alignment pipeline with software, version, and options chosen (when non-default options used).

Purpose	Software version	Options
Genome data visualization, Karyotype construction, and scaffold and bundle filtering	JupiterPlot-1.0 circulize v0.4.15	ref = HetTit1.0 scaf = HetAmer1.0 m = 4,000,000 ng = 95 *t* = 24 ref = ioIscEleg1.2 scaf = HetTit1.0 m = 100,000 ng = 80 minBundleSize = 2,500 maxGap = 1000000 ref = ioIscEleg1.2 scaf = HetAmer1.0 m = 100,000 ng = 80 minBundleSize = 2,500 maxGap = 1,000,000
Pairwise scoring of alignment to calculate percentage of identity	LASTZ-1.04.15	lastz_32 --notransition --step = 20 --nogapped --progress = 1 --gfextend --chain --format = blastn

## Results

### Sequence output and genome assembly

**Fig. 2.**
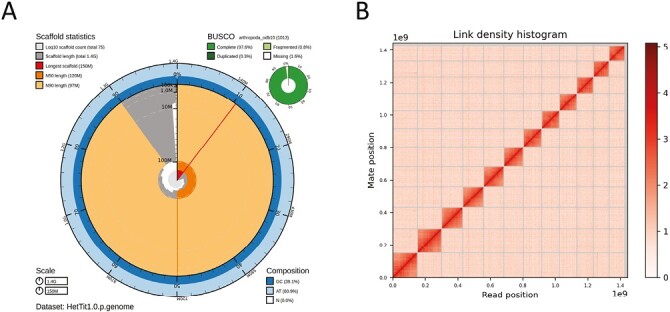
A) The graphical representation of the primary (i.e. principal pseudohaplotype) draft genome assembly produced by blobtools2. The main centre plot is divided into 1,000 size-ordered bins around the circumference with each bin representing 0.1% of the 1,444,697,970 bp assembly. The distribution of scaffold lengths is shown as blocks moving clockwise with the plot radius scaled to the longest record present in the assembly (151,479,759 bp, denoted by the first radial line). The N50 and N90 record lengths (120,343,728 and 97,068,442 bp) are denoted by the second and third radial lines, respectively. The spiral shows the cumulative record count on a log scale with dashed lines showing successive orders of magnitude. The outside of the plot shows the distribution of GC, AT, and N percentages in the same bins as the inner plot. A summary of complete, fragmented, duplicated, and missing BUSCO genes in the arthropoda_odb10 set is shown in the top right (see Table 3 for further details). B) The Hi-C sequencing contact map. The density of the positions of the paired Hi-C sequence reads mapped onto the scaffolds of the primary assembly.

Sequencing attained 3,268,207 total PacBio HiFi reads spanning 24,472,197,766 bp (17.4× coverage for a genome of 1.4 Gb, L50 = 8,304, N50 = 1068.5 kb). Omni-C sequencing generated 161,707,092 paired reads spanning 48,512,127,600 bp with an estimated coverage of 34.7× (for a genome of 1.4 Gb). Analyses in *genomescope* estimated a genome size of 1.33 Gb, a heterozygosity of 0.50%, and a sequencing error rate of 0.13%. K-mer density plots showed two peaks in coverage at ~8× and ~15× corresponding to the heterozygous and homozygous regions of the diploid genome. *HiFiAdapterFilt* v2.0 identified 581 PacBio HiFi reads as adapter contaminated which we removed, and the remaining reads were assembled into contigs using *Hifiasm*. All assembly statistics, as well as NCBI Accession Numbers, are provided in Table 3, and assembly quality is depicted graphically in Fig. 2a.

**Table 3.** Sequencing and assembly statistics and accession numbers. Including BioProjects and Vouchers for raw sequence reads and samples, genome assembly metrics calculated by *quast*/5.2.0, BUSCO scores calculated using arthropoda_ob10 and insecta_ob10 (C = complete, S = single, D = duplicated, F = fragmented, M = Missing), and the organelle assembly accession number. Sequencing read coverage based on a genome size of 1.4 Gb. Bp, base pairs; P, primary assembly; A, alternative assembly.

BioProjects and vouchers	Species NCBI BioProject	PRJNA906955					
	NCBI BioSample	SAMN32641599					
	Specimen ID	HXRCb13					
	NCBI Genome Accessions	Primary			Alternate		
	Genome sequences	JAVFHJ000000000			JAVFHK000000000		
Genome Sequencing	PacBio HiFi reads	Run			1 PACBIO_SMRT (Sequel II) run: 8M spots, 24.4 G bases, 17.05 Gb		
		Accession			SRR23023424		
	Omni-C data	Accession			SAMN35765994		
Genome Assembly Quality Metrics	Assembly identifier	HetTit1.0					
	HiFi read coverage	17.4×					
	Omic-C read coverage	34.7×					
		Primary			Alternate		
	No. contigs	4,094			4,054		
	Contig N50 (bp)	638,359			664		
	Contig L50	639			631,942		
	Longest contig	4,037,799			3,160,621		
	Number of scaffolds	75			NA		
	Scaffold N50	120,343,728			NA		
	Scaffold L50	6			NA		
	Largest scaffold	151,479,759			NA		
	Size of final assembly (bp)	1,444,296,070			1,429,703,052		
	BUSCO completeness (insect_odb10) *n* = 1,367		C	S	D	F	M
		P	97.0%	96.0%	1.0%	1.6%	1.4%
		A	95.9%	94.9%	1.0%	2.0%	2.1%
	BUSCO completeness (arthropoda_odb10) *n* = 1,013		C	S	D	F	M
		P	97.6%	97.3%	0.3%	0.8%	1.6%
		A	96.2%	95.7%	0.5%	1.3%	2.5%
Organelle	Mitochondrion	NCBI Accession Number					OQ363879

The total length of the scaffolded primary assembly was 1.44 Gb, which is inline with the estimate of 1.33 Gb from *genomescope*. The majority (98.8%) of the primary assembly was scaffolded into 12 chromosomal pseudomolecules. The final primary assembly consisted of 75 scaffolds, with a maximum scaffold length of 151.5 Mb, an L50 of 6, and an N50 of 120 Mb. There were 3798 gaps in the primary scaffolds with a total length of *N* bases of 0.4 Mb (0.02% of the total assembled scaffolds’ length). The alternative assembly was 1.43 Gb and consisted of 4,054 contigs with a maximum contig length of 3.1 Mb, an L50 of 664, and a N50 of 0.6 Mbps.

BUSCO analyses suggest that both primary and alternate pseudohaplotypes are near-complete genomes, with little duplication or missing data (Table 3). The primary assembly arthropoda BUSCO score was [C: 97.6% (S: 97.3%, D: 0.3%), F: 0.8%, M: 1.6%].

Our final circular mitochondrial assembly was 17,701 bp long, with a base composition of A = 31.0%, T = 40.5%, G = 15.7%, and C = 12.8%. Its annotation consisted of 22 transfer RNAs and 13 protein-coding genes.

**Fig. 3:**
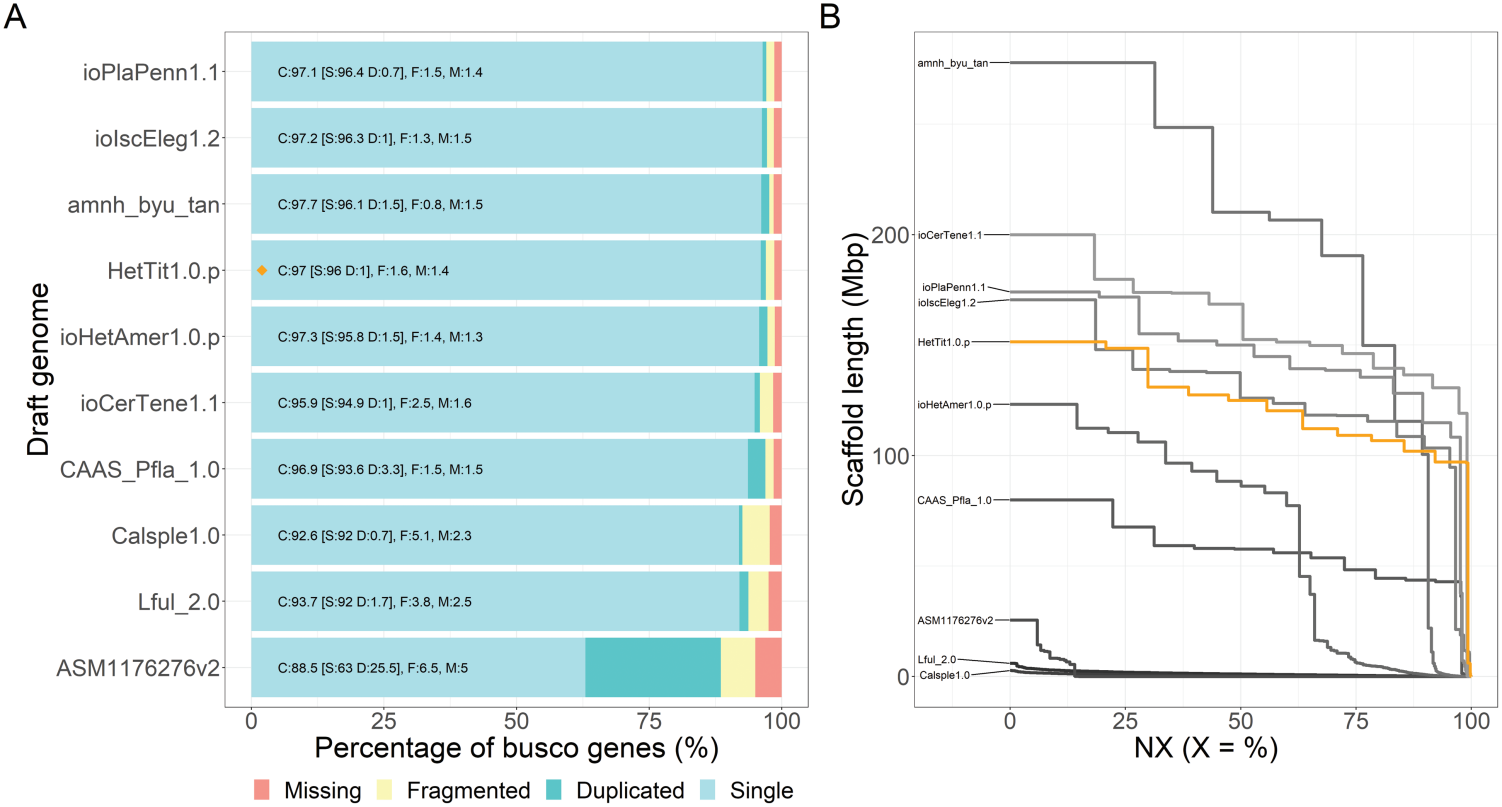
Comparison of the genome completeness and contiguity for the 10 odonate genomes available on GenBank, with the primary assembly for *Hetaerina titia* (HetTit1.0.p). A) BUSCO scores for each assembly using the insecta_ob10 dataset. Our genome assembly, HetTit1.0.p, is marked with a diamond. B) NGx plot showing scaffold/contigs of each assembly ordered from largest to smallest and the cumulative percentage of the genome covered. Included genomes are ioPlaPenn1.1, *Platycnemis pennipes* (Price et al. 2023), CAAS_Pfla_1.0, *Pantala flavescens* (Liu et al. 2022), ioSymStri1.1, *Sympetrum striolatum* (Crowley et al. 2023), ioCerTene1.1 *Ceriagrion tenellum* (GenBank: GCA_963169105.1), amnh_byu_tan, *Tanypteryx hageni* (Tolman et al. 2023), Lful_2.0, *Ladona fulva*, (GenBank: GCA_000376725.2), ioHetAmer1.0.p, *Hetaerina americana*, (Grether et al. 2023) Calsple1.0, *Calopteryx splendens* (Ioannidis et al. 2017), ASM1176276v2, *Rhinocypha anisoptera* (GenBank: GCA_011762765.2), and ioIscEleg1.2, *Ischnura elegans*, (Price et al. 2022).

BUSCO and NGx metrics show that our genome is among the most complete and contiguous odonate genomes available (Fig. 3).

### Comparative analyses

**Fig. 4.**
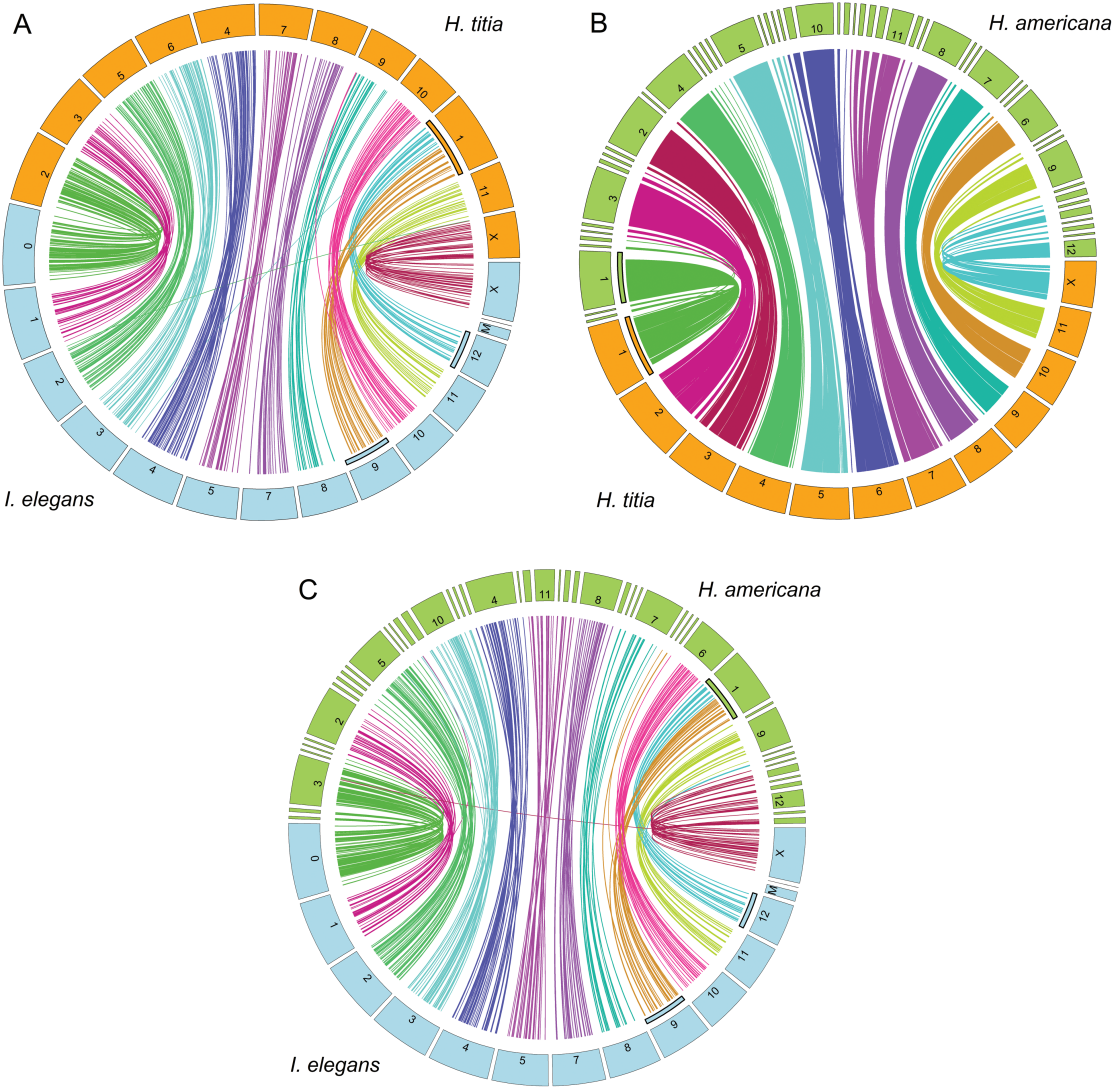
Circos assembly consistency plot showing the aligned regions between genome assemblies. Displayed scaffolds are restricted to those larger than 1 Mb. A) Scaffolds of *Ischnura elegans* (below) (Price et al. 2022) and *Hetaerina titia* (above), B) Scaffolds of *Hetaerina americana* (above) (Grether et al. 2023) and *H. titia* (below). C) Scaffolds of *H. americana* (above) and *I. elegans* (below). Internal highlighting of chromosomes indicates a fusion event between chromosomes 9 and 12 of *I. elegans* into chromosome 1 of *H. titia* and *H. americana*. Scaffolds for *H. titia* and *H. americana* are numbered by length, largest to smallest. The *H. titia* X chromosome was identified by mapping to the X chromosome of *I. elegans*. Chromosome numbers for *I. elegans* were retrieved from NCBI. Scaffold and chromosome labels are consistent between plots.

There was a high level of correspondence between *H. titia*, *H. americana*, and *I. elegans* genomes (Fig. 4a). However, neither *H. titia* nor *H. americana* had a corresponding m-chromosome, a comparatively small pair of autosomes found in *I. elegans* and other odonates. In addition, the upper and lower half of the largest scaffold within the *H. titia* and *H. americana* genome (scaffold 1) mapped to two different chromosomes of *I. elegans* (scaffolds 9 and 12).

There is a high level of correspondence with the reference genome of *H. americana* (Fig. 4b). *H. titia* scaffold 1, which matched two separate chromosomes of the *I. elegans* genome, mapped to a single large scaffold on the *H. americana* draft genome.

We calculated the overall similarity in aligned sequences between *H. titia* and *H. americana* as 88.44%. The overall similarity in aligned sequences between *H. titia* and *I. elegans* was 81.35%. The overall similarity between *H. americana* and *I. elegans* was 80.58%.

## Discussion

We generated a highly complete, high-quality de novo genome assembly from long-read and Omni-C sequence data obtained from two female smoky rubyspot damselfly (*H. titia*). BUSCO scores for the *H. titia* assembly reveal a more complete genome than many other existing assemblies of odonates. The highest single complete insecta BUSCO score, for another Odonata genome, is 97.4% for *Platycnemis pennipe*s (Price et al. 2023) vs. 97.0% for our draft genome of *H. titia* (for further comparison between all available odonate genomes (see Fig. 3a). The 12 largest scaffolds are longer than 100 Mbps, which is comparable to the lengths of chromosomes of other odonates (Fig. 3b, Ioannidis et al. 2017; Price et al. 2022, 2023), and comprise 98.8% of the total draft genome. Moreover, our mitochondrial assembly is highly complete and represents the first annotated mitochondrial assembly for *Hetaerina*. Our genome size estimate for *H. titia* (1.44 Gb) is larger than an earlier estimate based on microscopy (1.1 Gb—Ardila-Garcia and Gregory 2009) but smaller than the sequencing-based estimates for *H. americana* (1.63 Gb—Grether et al. 2023) and Calopteryx *splendens* (1.6 Gb—Price et al. 2022).

Previous estimates of *Hetaerina* karotypes, including *H. americana* and *H. titia,* were 2N = 24A + X (not including the m-chromosomes) (Kuznetsova and Golub 2020). Our findings suggest that the genome of *H. titia* is 2N = 22A + X, and does not have an m-chromosome, conflicting with previous estimates for the species and most other Calopterygidae genomes, such as *C. splendens,* which are 2N = 24A + X (plus an additional m-chromosomes) (Kuznetsova and Golub 2020; Kuznetsova et al. 2021). The scaffold size of the *H. titia* draft genome was comparable to the chromosome-scale scaffolds of *I. elegans* (Price et al. 2022). The *I. elegans* genome was constructed using PacBio HiFi sequencing and scaffolded to Hi-C data similar to that used in our genome assembly for *H. titia*. Each large scaffold of *H. titia* mapped concordantly onto a corresponding chromosome of *I. elegans*, with two exceptions: 1) The upper and lower half of scaffold one mapped onto two separate chromosomes of *I. elegans* and 2) there was no corresponding scaffold mapping to the m-chromosome of *I. elegans*. The linked density histogram of *H. titia* (Fig. 2a) clearly indicates that *H. titia* has a single large chromosome and contains no evidence of an m-chromosome. The mapping of the *H. titia* and *H. americana* genome to the *I. elegans* genome (Fig. 3a and c) supports a chromosomal fusion event and the loss of the m-chromosome in the shared ancestry of *H. titia* and *H. americana* making the karyotype for these species 2N = 22A + X. Nearly all damselflies have greater than 22 autosomes making a fusion, rather than a fission event, within the ancestry of *H. americana* and *H. titia* the most parsimonious explanation. The loss of the m-chromosome is common within damselflies and its presence is often inconsistent within cytogenetic studies of the same species (Kuznetsova and Golub 2020).

The relatively low similarity of the compared genomes of *H. titia* vs. *H. americana* (88.44%) likely results from the long time separating *H. americana* and *H. titia* from their most recent common ancestor (estimated at ~33 Mya by Standring et al. 2022). The considerable phylogenetic distance further supports the value of a high-quality reference genome for *H. titia*. Our de novo assembly for *H. titia* will serve to facilitate resequencing and genotyping of samples from this and other closely related *Hetaerina* species. The *H. titia* draft genome will enable further research into the evolutionary and demographic history of populations of *H. titia* in relation to the highly variable polyphenism of the species. The addition of a new chromosome-scale assembly for Odonata will allow for further understanding of genome evolution within this early branching insect lineage (Tolman et al. 2023). Further annotation of the *H. titia* genome would benefit the study of functional sequence evolution compared with *H. americana* and other congeners, opening the way for molecular evolutionary studies. The *H. titia* assembly described here, in combination with the recently assembled *H. americana* genome, make rubyspot damselflies a genome-enabled genus.

## Data Availability

Data generated for this project have been collated in NCBI BioProject PRJNA906955. The PacBio HiFi reads (with adapter indices and low-quality reads removed) for the sample (NCBI BioSample SAMN32641599) have been submitted to the NCBI Sequence Read Archive (SRR23023424). The Omni-C Illumina HiSeqX reads for the sample (NCBI BioSample SAMN35765994) have been submitted to the NCBI Sequence Read Archive (SRR25489624). The GenBank organelle assembly is submitted under the Accession number OQ363879. GenBank accessions for primary and alternate assemblies are JAVFHJ000000000 and JAVFHK000000000, respectively. (Note: we will make GenBank uploads available upon initial acceptance—reviewers can access these through this link: https://dataview.ncbi.nlm.nih.gov/object/PRJNA906955?reviewer = 9vq15drfs0d06cflstnkdcipdc). Assembly code is available on github: https://github.com/ChristophePatterson/Hetaerina_titia_genome.
